# Phenotypic and Functional Diversities of Myeloid-Derived Suppressor Cells in Autoimmune Diseases

**DOI:** 10.1155/2018/4316584

**Published:** 2018-12-23

**Authors:** Huijuan Ma, Chang-Qing Xia

**Affiliations:** ^1^Department of Nephrology, Jining No. 1 People's Hospital, Jining 272011, China; ^2^Department of Pathology, Immunology and Laboratory Medicine, University of Florida College of Medicine, Gainesville, FL 32610, USA

## Abstract

Myeloid-derived suppressor cells (MDSCs) are identified as a heterogeneous population of cells with the function to suppress innate as well as adaptive immune responses. The initial studies of MDSCs were primarily focused on the field of animal tumor models or cancer patients. In cancer, MDSCs play the deleterious role to inhibit tumor immunity and to promote tumor development. Over the past few years, an increasing number of studies have investigated the role of MDSCs in autoimmune diseases. The beneficial effects of MDSCs in autoimmunity have been reported by some studies, and thus, immunosuppressive MDSCs may be a novel therapeutic target in autoimmune diseases. There are some controversial findings as well. Many questions such as the activation, differentiation, and suppressive functions of MDSCs and their roles in autoimmune diseases remain unclear. In this review, we have discussed the current understanding of MDSCs in autoimmune diseases.

## 1. Introduction

Myeloid-derived suppressor cells (MDSCs) started to be described more than three decades ago mainly in cancer [1, 2]. The suppressive effects of MDSCs on immune responses lead to the failure of immune surveillance of cancer and promotion of tumor angiogenesis and metastasis. Thus, MDSCs are suggested to be an important cell component for creating tumor immunosuppressive microenvironment [3–7]. In recent years, it has been reported about the involvement of MDSCs in a variety of inflammatory disorders, including autoimmune diseases [8–12]. MDSCs serve as the negative regulator of immune responses, and they are likely to play a protective role in autoimmune diseases by inhibiting T cell-mediated immune responses. Most of the studies of MDSCs in autoimmunity are carried out in animal experiments, and some findings are controversial. The real biological and pathological roles of MDSCs in autoimmune diseases still need to be further characterized. In this review, we summarize the origin, phenotype, and functional characteristics of MDSCs and their involvement in autoimmune diseases as well as MDSCs as potential targets for therapeutic intervention.

## 2. Origin, Phenotype, and Functional Characteristics of MDSCs

Common myeloid precursor cells derive from hematopoietic stem cells (HSCs) in the bone marrow, and they give rise to “immature myeloid cells” (IMCs) without suppressive features in an unactivated state [13]. In healthy individuals, IMCs can differentiate into mature, functional dendritic cells (DCs), macrophages, and granulocytes [14]. However, in certain pathologic conditions, such as inflammation, tumors, infections, trauma, transplants, sepsis, or autoimmune diseases, the differentiation of IMCs is impaired, and subsequently, IMCs are activated and proliferate in response to diverse endogenous and exogenous factors [13, 15–17]. As a result, IMCs differentiate into MDSCs, resulting in the dramatic expansion and accumulation of a large number of MDSCs in peripheral tissues. MDSCs can potently inhibit immune responses through the expressions of suppressive factors [13].

In mice, MDSCs are characterized by the coexpression of CD11b and Gr-1. The CD11b^+^Gr-1^+^ cell population is divided into two relatively distinct subsets: M-MDSCs (CD11b^+^Ly6C^hi^Ly6G^−^) with monocytic morphology and G-MDSCs (CD11b^+^Ly6C^low^Ly6G^+^) with granulocytic morphology [18]. Recently, the expressions of CD115, CD80, CD124, F4/80, CD16, and CD31 have also been suggested as markers for identifying MDSCs, although these markers are not specific for MDSCs [19, 20].

Different from murine MDSCs, most human MDSCs express both CD11b and CD33 and have an absent or low expression of HLA-DR. Therefore, human MDSCs can be generally defined as CD11b^+^CD33^+^HLA-DR^low/-^. Within this population, monocytic MDSCs and granulocytic MDSCs can be further characterized by the phenotype of CD14^+^CD15^low/-^ and CD14^−^CD15^+^CD66b^+^, respectively, which seems to be consistent with hematologic morphology [13, 21]. Given the heterogeneity of MDSCs populations and the different combinations of markers used, there may be some overlap between the subsets of MDSCs, and these classifications are somewhat controversial [22, 23]. In a recent study, a high level of lectin-type oxidized LDL receptor 1 (LOX-1) was identified in polymorphonuclear MDSCs (PMN-MDSCs) in the peripheral blood and tumor tissues of cancer patients, which was associated with endoplasmic reticulum stress and lipid metabolism [24]. Lately, another study showed that the phenotypic and functional characteristics of MDSCs can shift at different clinical stages of multiple sclerosis (MS) [25].

MDSCs require different signals for their expansion and activation. A variety of factors play important roles in the expansion of MDSCs such as cyclooxygenase-2 (COX-2), prostaglandin E2 (PGE2), granulocyte/macrophage colony-stimulating factor (GM-CSF), macrophage colony-stimulating factor (M-CSF), IL-6, IL-3, vascular endothelial growth factor (VEGF), and stem cell factor (SCF)-1 [13, 26–29]. The activation of MDSCs is associated with IFN-*γ*, TGF-*β*, IL-13, IL-4, etc. [13]. These factors can trigger signaling pathways in MDSCs which are involved in regulating the processes of cell differentiation, proliferation, and apoptosis during hematopoiesis [30–32].

MDSCs can suppress the immune response through a variety of different mechanisms, including close cell-cell contact and soluble mediators, of which the predominant factors are arginase-1 (Arg-1) and inducible nitric oxide synthase (iNOS) [33]. The expressions of Arg-1 and iNOS which generate NO responsible for the MDSCs suppressive function are upregulated by activated MDSCs. The common substrate of Arg-1 and iNOS is L-arginine. Reactive oxygen species (ROS) represent another important mechanism [13, 34–37]. The monocytic MDSCs mainly associated with inflammation were found to express high levels of iNOS and low levels of ROS, whereas the granulocytic MDSCs mainly associated with tumors expressed high levels of ROS and low levels of iNOS [13]. Both subsets expressed Arg-1 [18, 38]. In addition, indoleamine 2,3-dioxygenase (IDO), IL-10, PGE2, COX-2, program death ligand 1 (PD-L1), and TGF-*β* also are very important to enable MDSCs to inhibit T cell proliferation and cytotoxicity [39–42]. MDSCs also facilitate regulatory T cells (Tregs) to exert suppressive functions. It has been shown that Gr-1^+^CD115^+^ MDSCs can promote the development of Foxp3^+^ Tregs in vivo and mediate the inactivation of tumor-specific T cells in a tumor mouse model [43]. Further studies are required to demonstrate whether MDSCs are involved and associated with Tregs in a common immune regulatory network. Moreover, a recent study found that Ly6G^+^ PMN-MDSCs could control the selective accumulation and cytokine secretion of B cells in the central nervous system (CNS), which facilitated the recovery of disease in experimental autoimmune encephalomyelitis (EAE) [44]. The relationship between MDSCs and B cells also remains to be further studied.

## 3. The Role of MDSCs in Autoimmunity

MDSCs' function has mostly been studied in animal tumor models and cancer patients. Recently, a growing body of evidence has suggested that MDSCs may be actively participating in the development of autoimmune diseases, such as MS [8, 25, 45], systemic lupus erythematosus (SLE) [11, 46], type 1diabetes (T1D) [47], inflammatory bowel disease (IBD) [9, 48], and rheumatoid arthritis (RA) [49]. However, the in vitro and the in vivo studies are sometimes controversial.

### 3.1. In Vitro Study of MDSCs

Generally speaking, in vitro, CD11b^+^Gr-1^+^ cells isolated from autoimmune inflammatory sites are able to inhibit T cell proliferation, typically through the participation of iNOS and Arg-1. In the autoimmune hepatitis (AIH) mouse model, the accumulation of CD11b^+^Gr-1^+^ myeloid cells was observed in the BALB/c Tgfb1^−/−^liver. And only the isolated Ly6C^hi^ subset was able to efficiently suppress CD4^+^ T cell proliferation in vitro by several different mechanisms, including NO, IFN-*γ*, and cell-cell contact [50]. Similarly, in the IBD mouse model, CD11b^+^Gr-1^+^ myeloid cells accumulated in the spleens and secondary lymphoid tissues, and only CD11b^+^Ly6C^hi^Ly6G^−^ MDSCs suppressed the proliferation and production of cytokines by CD4^+^ T cells, which were mediated by NO, cell-cell contact, and partially by IFN-*γ* and PGs [48]. It was shown that CD11b^+^Ly6C^hi^Ly6G^−^ MDSCs isolated from the spleen after EAE were induced potently inhibited CD4^+^ and CD8^+^ T cell proliferation, and induced apoptosis of proliferating T cell ex vivo, which was mediated by iNOS activity [8]. In line with these findings, MDSCs were also involved in experimental autoimmune uveoretinitis (EAU). These cells expressed CD11b phenotypically resembling monocytes and were accumulated in the inflamed eyes. In vitro, T cell proliferation could be greatly suppressed by these isolated monocyte-like cells [51]. Subsequent research in EAU showed that the intact TNF response axis was responsible for the suppressive function of MDSCs [52]. In line with the above reports, CD11b^+^Gr-1^low^ MDSCs were also identified in lupus-prone MRL-Fas^lpr^ mice that develop autoimmune organ damages. These cells had a suppressive effect on CD4^+^ T cell proliferation ex vivo, and Arg-1 inhibitor could block the suppression, indicating that arginase served as the dominant suppressive factor of MDSCs in this autoimmune setting [11]. Recently, in the pristane-induced lupus mouse model, we observed that CD11b^+^Ly6C^hi^ monocytes sorted from the peritoneal cells greatly inhibited T cell proliferation ex vivo which was mediated by cell-cell contact, NO, and PGE2 and could inhibit Th1 differentiation but enhanced the development of Tregs [53]. Our findings provide a novel insight into the role of Ly6C^hi^ monocytes mobilized by pristane injection in the pathogenesis of pristane-induced lupus in mice. We believe the Ly6C^hi^ monocytes induced by pristane injection are monocytic MDSCs.

### 3.2. In Vivo Study of MDSCs

Despite that MDSCs can potently inhibit T cell responses in vitro, the presence of MDSCs in autoimmune diseases is different, and current studies have shown conflicting roles for MDSCs in autoimmunity, either as an aggravating or as a curative factor of disease.

EAE is a common mouse model for multiple sclerosis, which is an autoimmune inflammatory neurological disease. Several studies have tried to demonstrate the possible role of MDSCs in EAE. King et al. observed that CD11b^+^CD62L^+^Ly6C^hi^ cells were mobilized increasingly and accumulated in the blood and CNS before clinical episodes of the disease, and these cells were subsequently matured into inflammatory macrophages and/or functional DCs. Thus, the study concluded that the accumulation of CD11b^+^Ly6C^hi^ monocytes in vivo served as pathologic effectors and was associated with EAE pathogenesis [45]. Similarly, the Mildner study showed that the selective depletion of CCR2^+^Ly6C^hi^ monocytes strongly reduced the CNS autoimmunity, indicating a disease-promoting role of CCR2^+^Ly6C^hi^ monocytes during autoimmune inflammation of the CNS [54]. Yi et al. also confirmed that the expansion of CD11b^+^Gr-1^+^ cells was present in the development of EAE. Although these MDSCs inhibited T cell proliferation, they promoted inflammatory Th17 cell differentiation in vitro mediated by IL-1*β*. Selective depletion of MDSCs using gemcitabine resulted in a marked reduction in the severity of EAE, and the adoptive transfer of MDSCs after this treatment restored EAE disease progression. The authors also demonstrated that the severity of EAE was correlated with the frequency of Th17 cells and the levels of inflammatory cytokines [55]. All the above findings in EAE indicate that MDSCs in vivo serve as pathologic effectors. However, some other studies had different conclusions about the activity of MDSCs. Ioannou et al. reported that before the disease remission, CD11b^hi^Ly6G^+^Ly6C^−^ granulocytic MDSCs were abundantly accumulated in the peripheral lymphoid organs. Adoptive transfer of G-MDSCs potently delayed the development of EAE through the suppressive effect on the priming of Th1 and Th17 cells. The upregulation of PD-L1 upon exposure to the autoimmune milieu both in vitro and in vivo was essential for the suppressive function of G-MDSCs [56]. Taken together, it seems that MDSCs have opposite roles in EAE, having both inflammatory functions and protective functions. The discrepancy of different reports suggests that further characterization of MDSCs in various autoimmune settings is needed. It is also possible more functionally diverse MDSCs subsets may exist.

Lately, MDSCs have been involved in the development of SLE associated with organ damages. A study reported that the deletion of CD24 in a lupus-like disease model driven by heat shock proteins (HSPs) led to the increase of CD11b^+^Gr-1^+^ MDSCs and Tregs that augmented immune tolerance, accompanying with the alleviation of lupus-like renal pathology [57]. On the contrary, it was recently shown that in a humanized SLE model MDSCs contributed to induce Th17 responses and related renal damage which was dependent on Arg-1 [58]. In addition, a recent study demonstrated that there were gender differences about the cellular and functional characteristics of myeloid cells in (NZB×NZW) F1 mice. The greatly increased Gr-1^hi^Ly6G^+^CD11b^+^ myeloid cells in male mice were capable of inhibiting autoantibody production and IL-10 production and slowing the progression of lupus-like disease in vivo. Furthermore, the production of antinuclear autoantibodies was increased after anti-Gr-1 mAb treatment. In vitro Gr-1^hi^CD11b^+^ cells could directly inhibit B cell differentiation. The authors postulated that these cells represented an important inhibitory mechanism in male mice and involved in SLE pathogenesis [59]. In Roquin^san/san^ SLE mice, sorted MDSCs induced the expansion of IL-10-producing regulatory B cells in vitro via NO. After administration of MDSCs, the regulatory B cells in the spleens of Roquin^san/san^ mice were expanded but effector B cells were decreased, accompanied with the reduction of serum anti-dsDNA antibody levels and the improvement of renal pathology. Therefore, MDSCs were likely to be a promising therapeutic target in the pathogenesis of SLE [46]. In our in vivo experiment, the transfer of purified CD11b^+^Ly6C^hi^ pristane-induced peritoneal monocytes was able to greatly inhibit anti-keyhole limpet hemocyanin (KLH) antibody production induced by KLH immunization [53], suggesting that these cells may have a protective effect in chronic autoimmune inflammation in pristane-induced lupus.

The role for MDSCs in T1D has been recently studied. The expanded Gr-1^+^CD11b^+^ MDSCs induced by anti-CD20 treatment in a mouse model of diabetes were found to suppress T cell proliferation dependent on NO, IL-10, and cell-cell contact and induce Tregs differentiation via TGF-*β*. In vivo, the transient expansion of MDSCs induced by anti-Gr-1 treatment delayed the development of disease in NOD mice. These findings suggested that Gr-1^+^CD11b^+^ MDSCs contributed to establish immune tolerance and could be a novel immunotherapeutic target for T1D [47]. A recent study found that CD11b^hi^Gr-1^int^ MDSCs were significantly increased in the peripheral blood of diabetic NOD mice. The authors suggested that the expansion of MDSCs was involved in the onset of diabetes [60]. Another study demonstrated that the adoptive transfer of MDSCs had an Ag-specific suppressive function and could prevent the onset of T1D through the induction of CD4^+^CD25^+^Foxp3^+^ Tregs development and anergy in autoreactive T cells [61].

MDSCs were also described in other autoimmune diseases. In the mouse model of IBD induced in VILLIN-hemagglutinin (HA) transgenic mice, the significantly increased CD11b^+^Gr-1^+^ MDSCs in the spleen and intestine were found to induce T cell apoptosis and suppress T cell proliferation ex vivo in a NO-dependent manner as well. Furthermore, the isolated CD11b^+^Gr-1^+^ MDSCs inhibited T cell-mediated colitis in VILLIN-HA mice [9]. The isolated granulocytic MDSCs from the spleens in a collagen-induced arthritis (CIA) mouse model were found to inhibit CD4^+^ T cell proliferation in vitro. Moreover, these cells could suppress the differentiation of CD4^+^ T cells into Th17 cells. Adoptive transfer of MDSCs reduced the severity of joint inflammation in vivo, and the removal of MDSCs worsened the disease [49]. Another recent study found that CD11c^−^CD11b^+^GR-1^+^ MDSCs separated from the peripheral blood and spleens of CIA mice could inhibit T cell proliferation in vitro partly via IL-10 and Arg-1, and in vivo infusion of MDSCs significantly ameliorated rheumatoid inflammation [62]. Alopecia areata is an autoimmune skin disease, the characteristic of which is inflammatory immune responses that cause hair loss. In a mouse model of alopecia areata, Gr-1^+^CD11b^+^ MDSCs were capable of inhibiting T cell proliferation in vitro, and subsequent in vivo application led to partial restoration of hair growth [63]. MDSCs were also described in a mouse model of experimental autoimmune myasthenia gravis (EAMG), in which the adoptive transfer of MDSCs was found to effectively reverse the disease progression [64]. Further analysis showed that in MDSCs-treated EAMG mice, acetylcholine receptor- (AChR-) specific immune responses were suppressed, serum anti-AChR IgG levels were decreased, and complement activation was reduced, in which various immune-modulating factors, such as PGE2, iNOS and arginase, were actively involved [64].

Up to date, almost all studies about MDSCs in human focus on cancer. There are few about MDSCs in patients with autoimmune diseases. A study performed in T1D patients has shown that in peripheral blood mononuclear cells (PBMC) the frequency of CD11b^+^CD33^+^ MDSCs is significantly increased, but these MDSCs are not maximally suppressive in function, suggesting that functional defects in MDSCs may contribute to T1D pathogenesis [60]. Recently, a study on human SLE demonstrated the pathogenic role of MDSCs. Compared to healthy controls, HLA-DR^−^CD11b^+^CD33^+^ MDSCs in the peripheral blood of active SLE patients significantly increased. A positive correlation between the frequency of MDSCs and Th17 responses, serum Arg-1 level, and disease severity was observed, which provided new insights into the molecular mechanism targeting MDSCs for the treatment of SLE [58]. This increase of HLA-DR^−^CD11b^+^CD33^+^ MDSCs may be mobilized and recruited during the active inflammatory process in SLE, because inflammation can lead to myelopoiesis [65] potentially giving rise to these intermediate stages of myeloid cells. Additionally, the numbers of MDSCs in the peripheral blood and plasma Arg-1 level were greatly increased in RA patients. The elevated frequency of Th17 cells in those patients was observed to be negatively correlated with the plasma Arg-1 level and the frequency of MDSCs. It was also found that there was a negative correlation between the level of plasma TNF-*α* and MDSCs frequency [66]. Recently, another study about RA patients has shown that the expansion of MDSCs as a risk factor was associated with disease activity and joint inflammation [67]. Given the important role of MDSCs in modulating immune response, more research needs to be carried out to explore the effect of MDSCs in human autoimmune disorders.

In conclusion, MDSCs possess a variety of activities in autoimmune models and diseases (summarized in Table 1); it is therefore a challenge to draw a definitive conclusion on the roles of MDSCs in autoimmune diseases [68, 69]. Generally speaking, in vitro, the isolated CD11b^+^Gr-1^+^ MDSCs from inflammatory sites inhibit T cell responses dependent on various mechanisms such as NO and Arg-1. However, in vivo, endogenous MDSCs may be proinflammatory and fail to effectively reduce the severity of autoimmune diseases in several systems (e.g. in EAE [45, 54, 55]). By contrast, the adoptive transfer of MDSCs is able to induce immune tolerance to self-Ag and limits autoimmune pathology and has a beneficial effect on the autoimmune disease, as observed in models of IBD [9], T1D [61], and inflammatory eye disease [70]. The reasons that lead to these discrepancies between the activities of endogenous and exogenous MDSCs remain unclear. A possible explanation may be that certain factors coexisting in the same inflammatory microenvironment inhibit the suppressive activity of MDSCs, and the isolation of MDSCs is liberated from this “inhibitory” environment and MDSCs regain their immunosuppressive function upon readministration or addition into in vitro culture systems.

## 4. Therapeutic Potential of MDSCs in Autoimmune Diseases

The application of MDSCs exogenously in certain animal models shows great efficacy in suppressing autoimmune diseases, indicating that MDSCs might be a promising cellular immunotherapeutic target in autoimmune diseases. Several potential cellular sources are available for in vitro generated MDSCs. Exogenous MDSCs isolated from the peripheral blood or bone marrow could be markedly expanded in vitro by use of growth factor/cytokine regimens [71–73]. In addition, it has been reported that exogenous MDSC populations also can derive from hematopoietic stem cells and embryonic stem cells [74]. More recently, the monocytes isolated from the peripheral blood were cultured in vitro supplemented with PGE2, for the generation of high numbers of MDSCs, and their functional stability was established [75]. In vitro generated MDSCs share many characteristics with their ex vivo isolated counterparts. They have strong suppressive effect on the proliferation of CD4^+^ and CD8^+^ T cells mediated by the expressions of iNOS and/or Arg-1 and cell-cell contact [72]. All these methods above will be able to provide reliable cellular products for immunotherapy in treating autoimmune diseases. Several groups have shown that the adoptive transfer of ex vivo generated MDSCs had the ability to inhibit graft-versus-host disease (GVHD) and prevent allograft rejection in mice [73, 74, 76]. Meanwhile, there are some potential risks associated with the utilization of MDSCs to treat autoimmune diseases. For example, MDSCs utilized would be nonspecific for antigen-specific T cells. Therefore, the suppressive effects of MDSCs on harmful T cell responses for autoantigens and the protective immune responses to pathogenic microorganisms or tumors exist at the same time. It would also be difficult to control the migration and accumulation of the injected MDSCs. Additionally, the release of inflammatory factors may occur after the administration of MDSCs. Some other unpredictable risks may also exist. Thus, more extensive research in animal models is indispensable before MDSCs therapy moves into clinical studies.

## 5. Concluding Remarks

MDSCs are a highly heterogeneous cell subpopulation. They have multifaceted phenotypic characteristics and may suppress T cell proliferation through various mechanisms. Large numbers of factors are involved in the differentiation, migration, expansion, and activation of MDSCs. However, there are many unresolved questions in the field of MDSCs research. Up to now, the biological roles of MDSCs are rarely known. The role of endogenous MDSCs in autoimmune diseases in vivo remains controversial. Many questions about the variety of activities for MDSCs remain to be elucidated. It is necessary to understand why the induction and suppressive mechanisms of MDSCs are different between in vivo and in vitro environments. A better comprehension of the role of human MDSCs in autoimmunity and how to manipulate this cell population in patients with autoimmune diseases will be of great clinical significance. Importantly, exogenously prepared MDSCs have a great potential to become an effective immunotherapeutic regimen for autoimmune diseases.

## Figures and Tables

**Table 1:** Myeloid-derived suppressor cells in autoimmune diseases.

Disease	Species	Phenotype	Mechanism of suppression (in vitro)	Effect in vivo	Reference
Multiple sclerosis	Mouse	CD11b^+^Ly6C^hi^Ly6G^−^	NO apoptosis	Not determined	[8]
	Mouse	CD11b^+^CD62L^+^Ly6C^+^	Undetermined	Proinflammatory	[45]
	Mouse	CCR2^+^CD11b^+^Ly6C^hi^	Unknown	Increase severity	[54]
	Mouse	CD11b^+^Gr-1^+^	IL-1*β*	Increase severity	[55]
	Mouse	CD11b^hi^Ly6G^+^Ly6C^−^	PD-L1	Reduce severity	[56]

Systemic lupus erythematosus	Mouse	CD11b^+^Gr-1^low^	Arginase-1	Not determined	[11]
	Mouse	CD11c^−^CD11b^+^Gr-1^+^	NO	Suppressor	[46]
	Mouse	CD11b^+^Ly6C^hi^	NO, PGE2, cell-cell contact	Possibly protective	[53]
	Human	HLA-DR^−^CD11b^+^CD33^+^	Unknown	Increase severity	[58]
	Mouse	Gr-1^hi^Ly6G^+^CD11b^+^	ROS, NO	Suppressor (in males)	[59]

A lupus-like disease driven by HSP	Mouse	CD11b^+^Gr-1^+^	Unknown	Reduce severity	[57]

Type 1 diabetes	Mouse	CD11b^+^Gr-1^+^	NO, IL-10, cell-cell contact	Reduce severity	[47]
	Mouse	CD11b^hi^Gr-1^int^	Cell-cell contact	Proinflammatory	[60]
	Human	HLA-DR^−^CD11b^+^CD33^+^	Cell-cell contact	Proinflammatory	[60]
	Mouse	Gr-1^+^CD115^+^	MHC class II-restricted Ag presentation	Reduce severity	[61]

Inflammatory bowel disease	Mouse	CD11b^+^Gr-1^+^	NO apoptosis	Reduce severity	[9]
	Mouse	CD11b^+^Ly6C^hi^Ly6G^−^	NO, cell-cell contact, partially IFN-*γ*, PGs	Not determined	[48]

Rheumatoid arthritis	Mouse	CD11b^+^Ly6G^+^Ly6C^low^	Arg-1, NO	Reduce severity	[49]
	Mouse	CD11c^−^CD11b^+^GR-1^+^	IL-10, Arg-1	Reduce severity	[62]
	Human	HLA-DR^−^CD11b^+^CD33^+^	Unknown	Suppressor	[66]
	Human	CD11b^+^CD33^+^HLA-DR^−^	Unknown	Increase severity	[67]

Inflammatory eye disease	Mouse	CD11b^+^Gr-1^+^Ly6G^−^	TNFR-dependent, arginase	Not determined	[51, 52]
	Mouse	CD11b^+^Gr-1^+^	IL-6	Reduce severity	[70]

Autoimmune hepatitis	Mouse	CD11b^+^Ly6C^hi^Ly6G^−^	NO, IFN-*γ*, cell-cell contact	Not determined	[50]

Alopecia areata	Mouse	CD11b^+^Gr-1^+^	T cell apoptosis	Possibly protective	[63]

Experimental autoimmune myasthenia gravis	Mouse	CD11b^+^Gr-1^+^	PGE2, NO, Arg-1	Reduce severity	[64]
